# Hypoxia-inducible factor 1 alpha is regulated by RBM38, a RNA-binding protein and a p53 family target, via mRNA translation

**DOI:** 10.18632/oncotarget.2786

**Published:** 2014-11-16

**Authors:** Seong-Jun Cho, I-Fang Teng, Min Zhang, Tiffany Yin, Yong-Sam Jung, Jin Zhang, Xinbin Chen

**Affiliations:** ^1^ Comparative Oncology Laboratory, Schools of Medicine and Veterinary Medicine, University of California, Davis, CA

**Keywords:** p53, RBM38, RNPC1, HIF1α, mRNA translation

## Abstract

Hypoxia-inducible factor 1 (HIF1), a heterodimeric transcription factor, consists of HIF1α and HIF1β and is necessary for cell growth and survival under a hypoxic condition. Thus, the level and activity of HIF1α needs to be tightly controlled. Indeed, HIF1α protein stability is controlled by prolyl hydroxylase and von Hippel-Lindau-mediated proteosomal degradation. However, it remains unclear whether HIF1α expression is controlled by other pathways. Here, we showed that RNA-binding protein RBM38, a target of the p53 family, regulates HIF1α expression via mRNA translation. Specifically, we showed that under a hypoxic condition, ectopic expression of RBM38 decreased, whereas knockdown of RBM38 increased, the level of HIF1α protein. We also showed that the rate of de novo HIF1α protein synthesis was increased by knockdown of RBM38. Additionally, we showed that RBM38 directly bound to HIF1α 5′ and 3′UTRs. Consistently, we showed that the rate of mRNA translation for a heterologous reporter that carries HIF1α 5′and/or 3′UTRs was increased upon knockdown of RBM38. Furthermore, we showed that knockdown of RBM38 increased, whereas ectopic expression of RBM38 decreased, the binding of eIF4E to HIF1α mRNA. Together, our data suggest that RBM38 is a novel translational regulator of HIF1α under a hypoxic condition.

## INTRODUCTION

Hypoxia (low oxygen tension) induces an array of cellular processes to maintain ATP production via glycolysis and other survival pathways [1, 2]. Hypoxia-inducible factor 1 (HIF1), a well-defined hypoxia responsive factor, consists of two distinct subunits, HIF1 alpha (HIF1α) and HIF1β (ARNT). HIF1 belongs to a subfamily of the basic-helix-loop-helix-PAS transcription factors [3]. In response to high levels of oxygen, HIF1α protein is modified by prolyl hydroxylase and rapidly degraded through the VHL-mediated proteasomal pathway [1]. Once normoxia turns into hypoxia, prolyl hydroxylase is inactivated and subsequently, HIF1α is rapidly stabilized through decreased degradation [3]. Upon accumulation, HIF1α induces an array of target genes associated with cell survival (insulin-like growth factor-binding protein-1, Nip3), angiogenesis (vascular endothelial growth factor A, angiopoietin-2, plasminogen activator inhibitor-1), and energy metabolism (glucose transporter-1, hexokinase-2, glyceraladehyde-3-phosphate dehydrogenase) [1, 3]. Additionally, HIF1α appears to possess transcription-independent activities through physical interaction with c-Myc and Cdc6 to regulate the cell cycle [4-6].

RBM38, also called RNPC1, is a target of the p53 family and a RNA recognition motif (RRM)-containing RNA binding protein [7]. RBM38 is expressed primarily as RBM38 (239 amino acids) along with a minor isoform, RBM38b (121 amino acids). RBM38b has a sequence identical to the N-terminal region of RBM38. RBM38 is known to regulate mRNA translation of p53 and mRNA stability of p21, HuR, p63, p73, MDM2, and MIC-1 transcripts [7-13].

Although HIF1α expression is mainly regulated by post-translational modifications and protein stability [3, 14], other pathways have been found to regulate HIF1α expression, including transcription and translation [15]. In this study, we showed that ectopic expression of RBM38 decreased, whereas knockdown of RBM38 increased, the level of HIF1α protein under a hypoxic condition. Moreover, we found that knockdown of RBM38 enhanced HIF1α mRNA translation via binding to HIF1α 5′ and 3′UTRs. Together, we uncovered a novel mechanism by which HIF1α is regulated by the p53 pathway via RBM38.

## RESULTS

### HIF1α expression is regulated by RBM38 under a hypoxic condition

**Figure 1:**
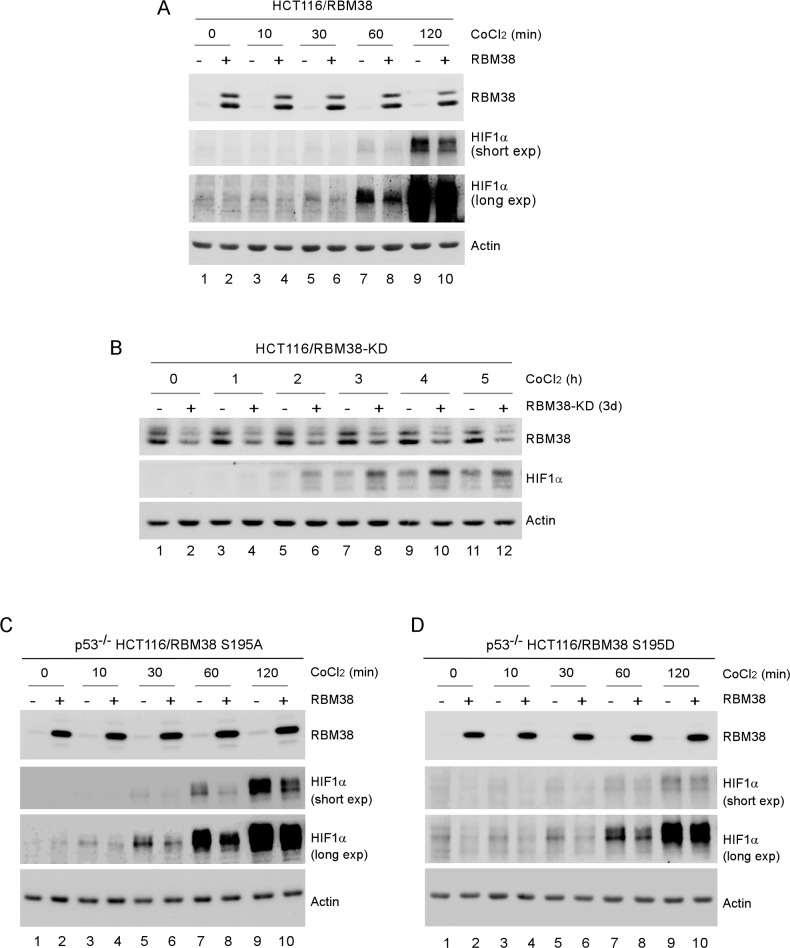
HIF1α expression is regulated by RBM38 under a hypoxic condition (A) HCT116 cells were uninduced (−) or induced (+) to express RBM38 with doxycycline for 24 h and then treated with 500 μM CoCl_2_ for the indicated times. The levels of RBM38, HIF1α, and actin were measured by Western blotting. (B) HCT116 cells were uninduced (−) or induced (+) to knock down RBM38 with doxycycline for 3 days and then treated with 500 μM CoCl_2_ for the indicated times. The levels of RBM38, HIF1α, and actin were measured by Western blotting. (C-D) p53^−/−^ HCT116 cells were uninduced (−) or induced (+) to express RBM38-S195A (C) or RBM38-S195D for 48 h and then treated with 500 μM CoCl_2_ for the indicated times. The experiments were performed as in (A).

HIF1α is necessary for cell survival under a hypoxic condition and its expression is controlled by multiple positive and negative regulators in addition to VHL-mediated proteasomal degradation [16]. Since HIF1α has a long 3′UTR along with an AU-rich element (ARE), we examined whether HIF1α expression is modulated by RNA-binding protein RBM38, a target of the p53 family and a potent regulator of multiple pro-survival and pro-death factors [7-13]. To test this, HCT116 cell line in which RBM38 can be inducibly expressed under the control of a tetracycline-regulated promoter was used. We showed that the level of HIF1α protein was decreased by RBM38 in HCT116 cells treated with CoCl_2_, a hypoxia mimetic (Fig. 1A). Next, we examined whether knockdown of RBM38 has an opposite effect on HIF1α expression under a hypoxic condition. To test this, HCT116 cell line in which RBM38 can be inducibly knocked down under the control of a tetracycline-regulated promoter was used. We found that under a hypoxia-mimic condition (CoCl_2_ treatment), the levels of HIF1α protein were increased by knockdown of RBM38 in a time-dependent manner (Fig. 1B).

Previously, we showed that phosphorylation of RBM38 modulates RBM38 to regulate p53 expression. To test this, the effect of phosphorylation of RBM38 on HIF1a expression was measured in p53-null HCT116 cells, which can inducibly express RBM38-S195A, a non-phosphorylatable form, or RBM38-S195D, a phosphor-mimetic. We found that under a hypoxia-mimic condition (CoCl_2_ treatment), the levels of HIF1α protein were decreased by both RBM38-S195A and RBM38-S195D (Fig. 1C-D), suggesting that RBM38 is capable of regulating HIF1α regardless of its phosphorylation status.

**Figure 2:**
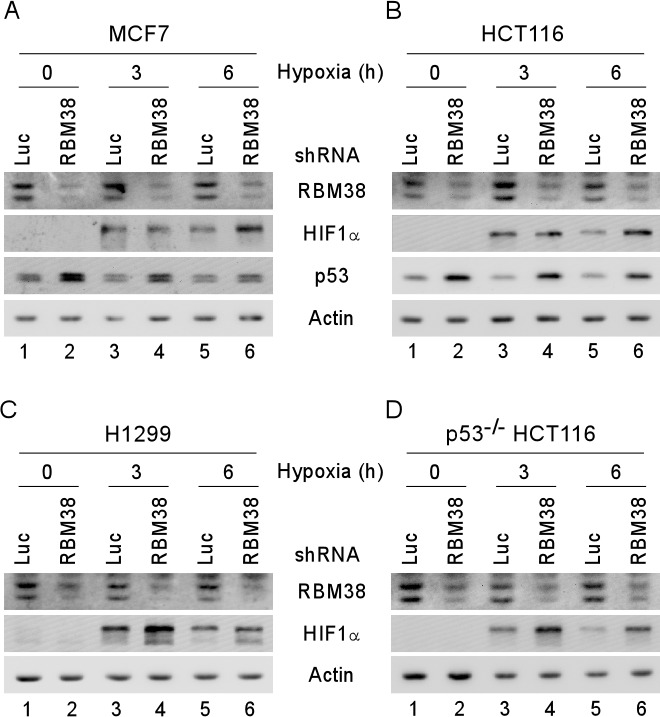
HIF1α expression is increased by knockdown of RBM38 under a hypoxic condition MCF7 (A), HCT116 (B), H1299 (C), and p53^−/−^ HCT116 (D) cells were transduced with a lentivirus expressing a control luciferase (Luc) shRNA or RBM38 shRNA, selected by puromycin for 3 d, and then exposed to hypoxia for 0, 3, or 6 h. Whole cell lysates were collected and the levels of RBM38, HIF1α, p53, or actin were determined by Western blot analysis.

To confirm the regulation of HIF1α by RBM38 under a hypoxic condition, MCF7 and HCT116 cells were transduced with a lentivirus expressing RBM38 shRNA or luciferase shRNA for 3 d and then incubated under a hypoxia condition (~0.1% oxygen) for various times. As a control, the levels of p53 protein were measured and found to be increased by knockdown of RBM38 regardless of the condition of oxygen tension (Fig. 2A-B, p53 panels), consistent with our previous studies [9, 17]. Interestingly, we found that the levels of HIF1α in both MCF7 and HCT116 cells were increased upon knockdown of RBM38 under a hypoxic condition for 6 h, but little if any under the same condition for 3 h (Fig. 2A-B, compare lanes 3 and 5 with lanes 4 and 6, respectively). Since p53 is capable of destabilizing HIF1α protein through the ubiquitin-dependent proteasomal degradation pathway [9, 14], p53-null HCT116 and H1299 cells were used to rule out potential effects of wild-type p53 on RBM38-mediated HIF1α regulation. Indeed, we found that the levels of HIF1α protein were markedly increased by knockdown of RBM38 under the same hypoxic condition for both 3 and 6 h (Fig. 2C-D, compare lanes 3 and 5 with 4 and 6, respectively). Together, these data suggest that RBM38 is necessary for maintaining proper expression of HIF1α under a hypoxic condition.

### RBM38 regulates HIF1α mRNA translation

**Figure 3:**
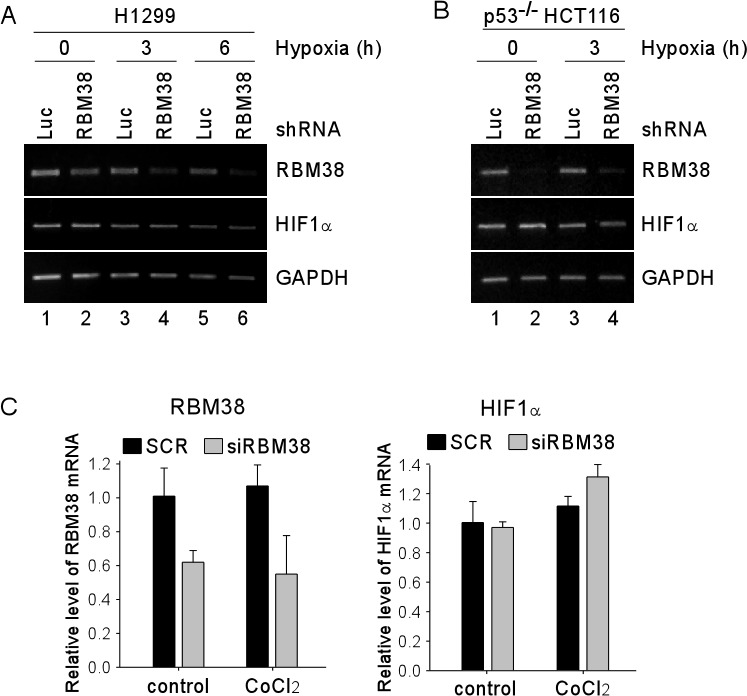
Knockdown of RBM38 has no effect on the level of HIF1α mRNA (A-B) H1299 (A) and p53^−/−^ HCT116 (B) cells were transduced with a lentivirus expressing a control luciferase (Luc) shRNA or RBM38 shRNA for 3 d, followed by exposure to hypoxia for 0-6 h for H1299 (A), and 0-3 h for p53^−/−^ HCT116 (B) cells. Total RNAs were isolated and RT-PCR was performed to measure the levels of RBM38, HIF1α, and GAPDH transcripts. (C) H1299 cells were transiently transfected with scramble siRNA (SCR) or siRNA against Rbm38 (SiRBM38) for 48 h, followed by treatment with 500 μM CoCl_2_ for 3 h. Total RNAs were isolated and quantitative RT-PCR was performed in triplicates to measure the levels of RBM38, HIF1α, and GAPDH transcripts. The levels of RBM38 and HIF1α transcripts were normalized to that of the GAPDH transcript. The relative fold change for RBM38 (left panel) and for HIF1α (right panel) is the ratio of the transcript level in cells with knockdown of RBM38 versus that in cells transfected with a control scrambled siRNA.

**Figure 4:**
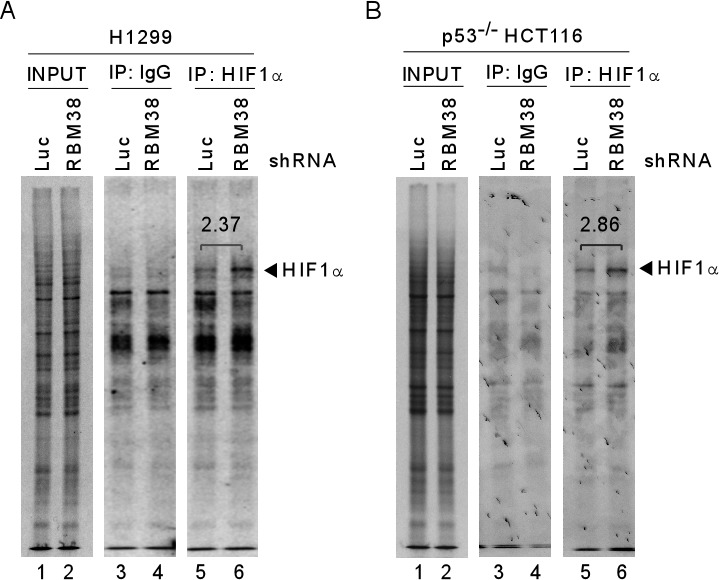
Knockdown of RBM38 enhances HIF1α expression through mRNA translation ^35^S-metabolic labeling assay was performed with H1299 (A) or p53^−/−^ HCT116 (B) cells. Cells were transduced with a lentivirus expressing a control luciferase (Luc) shRNA or RBM38 shRNA, selected by puromycin for 3 d, and then treated with 500 μM CoCl_2_ for 3 h, followed by labeling with ^35^S-methionine and ^35^S-leucine. Cell lysates were isolated and used for immunoprecipitation with anti-HIF1α (H1α67, Sigma) or non-immune mouse IgG. The samples from immunoprecipitation were separated in 8% SDS/PAGE and the protein signals were captured by autoradiography.

As an RNA-binding protein, RBM38 is known to regulate gene expression through post-transcriptional mechanisms, including mRNA stability and translation [9]. To explore how RBM38 regulates HIF1α expression under a hypoxic condition, RT-PCR was performed to measure the level of HIF1α transcript in H1299 and p53^−/−^ HCT116 cells upon knockdown of RBM38. We showed that the levels of RBM38 transcript were decreased by shRNA against RBM38 in H1299 and p53^−/−^ HCT116 cells exposed to hypoxia for various times (Fig. 3A-B). However, the levels of HIF1α transcript were not significantly altered by knockdown of RBM38 under both normoxic and hypoxic conditions (Fig. 3A-B). Similarly, under a hypoxia-mimic condition, the levels of HIF1α transcript were not significantly altered by knockdown of RBM38 in H1299 cells (Fig. 3C). Thus, we postulate that RBM38 regulates HIF1α expression potentially through mRNA translation. To test this, we measured the levels of newly synthesized HIF1α protein in ^35^S-labeled H1299 and p53^−/−^ HCT116 cells treated with CoCl_2_ for 3 h. Indeed, we found that the levels of newly synthesized HIF1α protein were markedly increased (2.37 and 2.86 fold) by knockdown of RBM38 in H1299 and p53^−/−^ HCT116 cells (Fig. 4A-B). Together, these data suggest that RBM38 regulates HIF1α mRNA translation under a hypoxic condition.

### RBM38 directly binds to HIF1α transcript

**Figure 5:**
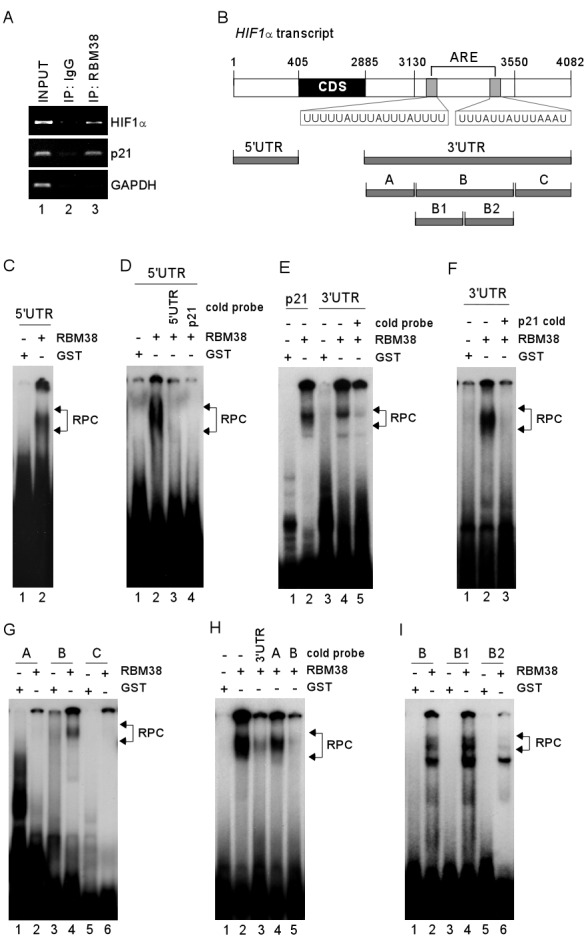
RBM38 directly binds to HIF1α 5′ and 3′ UTRs (A) Whole cell lysates from H1299 cells were collected and immunoprecipitated with anti-RBM38 antibody or control IgG, followed by RT-PCR to determine the level of HIF1α transcripts in control IgG and anti-RBM38 immunocomplexes. The levels of p21 and GAPDH transcripts were measured as positive and negative controls, respectively. (B) Schematic presentation of HIF1α transcript and the location of probes. Putative ARE regions are shown in shaded box. (C) REMSA was performed by mixing ^32^P-labeled RNA probe, HIF1α 5′UTR, with recombinant GST or GST-fused RBM38. (D) REMSA assay was performed by mixing ^32^P-labeled RNA probe, HIF1α 5′UTR, along with or without an excess amount (50-fold) of unlabeled HIF1α 5′UTR or p21 probe. (E-F) REMSA was performed by mixing ^32^P-labeled RNA probe (p21 or HIF1α 3′UTR probe) with recombinant GST or GST-fused RBM38 along with or without an excess amount (50-fold) of unlabeled HIF1α 3′UTR (E) or p21 probe (F). (G) REMSA was performed by mixing ^32^P-labeled RNA probe (fragment A, B, or C) with recombinant GST or GST-fused RBM38. (H) REMSA was performed as described in (F) except that cold HIF1α 3′UTR, fragment A, or fragment B was used. (I) REMSA was performed as described in (G) except that probes B, B1, and B2 were used. The arrow indicates RNA-protein complexes.

Considering that RBM38 is an RNA-binding protein, we postulate that the binding of RBM38 to HIF1α mRNA is required for regulating HIF1α expression. To test this, RNA immunoprecipitation was performed and showed that HIF1α mRNA was highly enriched in anti-RBM38-immunocomplexes, (Fig. 5A, HIF1α panel, compare lane 2 with 3). In addition, RBM38 was found to interact with p21 transcript (Fig. 5A, p21 panel), consistent with previous reports [7, 18]. In contrast, no interaction was found between GAPDH transcript and RBM38 (Fig. 5A). Next, a set of HIF1α RNA probes were generated and used for RNA Electrophoretic Mobility Shift Assay (REMSA) to map the binding sites of RBM38 in HIF1α transcript (Fig. 5B). We showed that recombinant RBM38 protein bound strongly to HIF1α 5′UTR (Fig. 5C) and 3′UTR (Fig. 5E, compare lanes 3-4). The binding of RBM38 to a probe derived from p21 3′UTR, which is known to carry a RBM38-response element [18], was performed and used as a positive control (Fig. 5E, compare lanes 1-2). To confirm the specificity of RBM38 binding to HIF1α transcript, RNA competition assay was performed and showed that the binding of RBM38 to HIF1α 5′UTR was abrogated by an excess amount of cold HIF1α 5′UTR or p21 probe (Fig. 5D, compare lanes 2 with 3-4, respectively). Similarly, the binding of RBM38 to HIF1α 3′UTR was abrogated by an excess amount of cold HIF1α 3′UTR (Fig. 5E, compare lanes 4-5) or cold p21 probe (Fig. 5F, compare lanes 2-3). To define the RBM38-binding site in HIF1α 3′UTR, three additional RNA probes, fragments A-C, were generated (Fig. 5B). We showed that RBM38 bound strongly to probe B, but not to A and C (Fig. 5G). Additionally, the binding of RBM38 to HIF1α 3′UTR probe was markedly inhibited by an excess amount of cold probe B and 3′UTR, but not by probe A (Fig. 5H, compare lanes 2 with 3-5, respectively). To further map the RBM38-binding site in fragment B, two sub-fragments, B1 and B2, were generated (Fig. 5B). We showed that RBM38 bound strongly to probes B and B1, but only weakly to B2 (Fig. 5I, compare lanes 1, 3, and 5 with 2, 4, and 6, respectively). These data suggest that RBM38 can directly bind to both HIF1α 5′ and 3′ UTRs.

### RBM38 regulates HIF1α mRNA translation through HIF1α 5′ and 3′ UTRs

**Figure 6:**
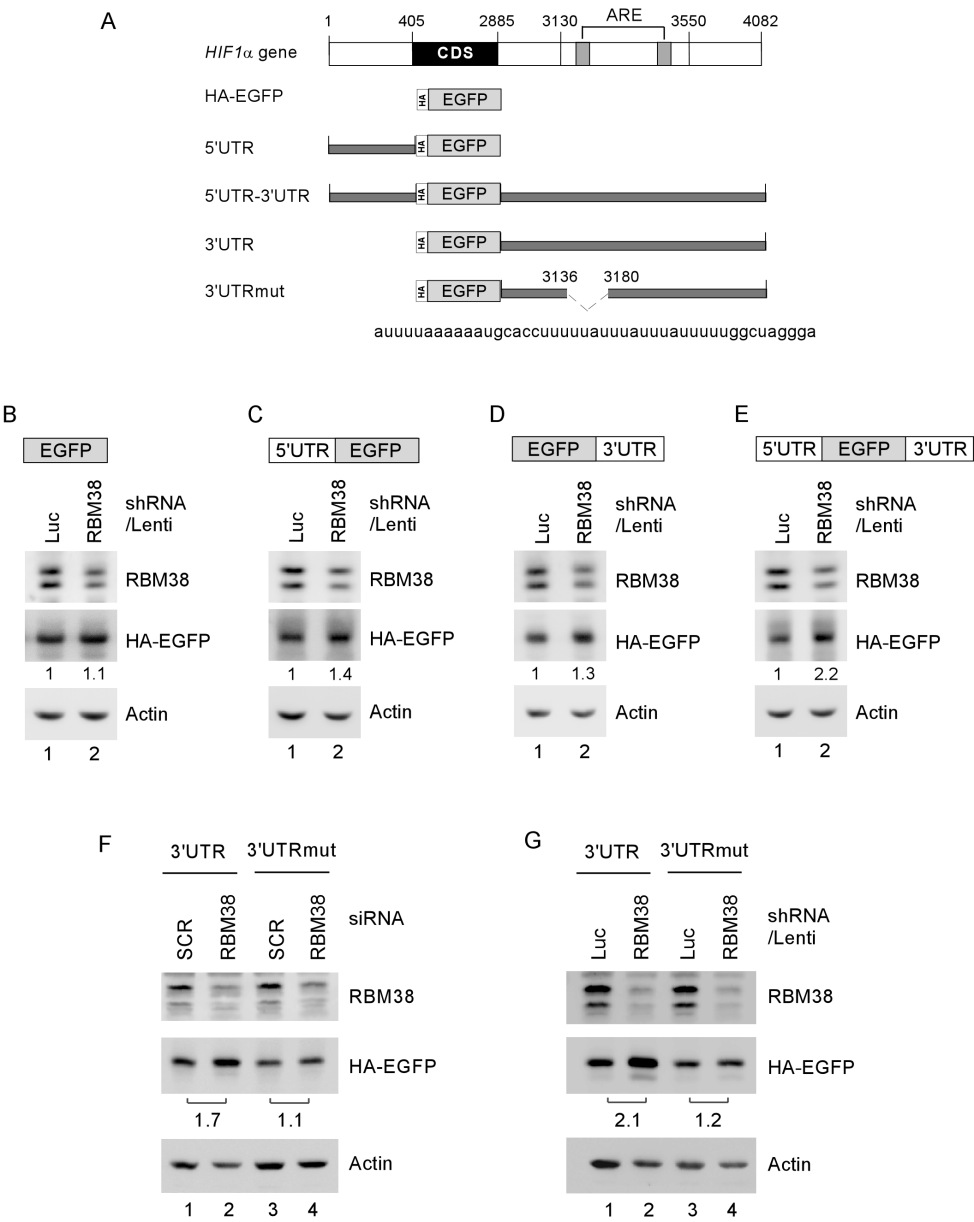
HIF1α 5′ and 3′UTRs are necessary and sufficient for RBM38 to regulate HIF1α expression (A) Schematic diagram of the HIF1α transcript and EGFP reporters along with HIF1a 5′ and/or 3′ UTRs. Deletion mutation in ARE region of HIF1α 3′UTR is shown in dash line. (B-E) p53^−/−^HCT116 cells were transduced with a lentivirus expressing control luciferase shRNA or RBM38 shRNAfor 48 h, and then transiently transfected with an EGFP expression vector that contains the coding region alone (B) or in combination with HIF1α 5′UTR (C), 3′UTR (D), or both (E). (F) p53^−/−^HCT116 cells were transfected with scramble siRNA (SCR) or siRNA against RBM38 for 3 days, and then transiently transfected with an EGFP expression vector that contains HIF1α 3′UTR or HIF1α 3′UTRmut. (G) p53^−/−^HCT116 cells were transduced with a lentivirus expressing control luciferase shRNA or RBM38 shRNA, selected by puromycin for 3 days and then transiently transfected with an EGFP expression vector that contains HIF1α 3′UTR or HIF1α 3′UTRmut. Whole cell lysates were collected and the levels of RBM38, HA-EGFP, and actin were determined by Western blot analysis.

To determine whether HIF1α 5′ and/or 3′UTRs are necessary and sufficient for RBM38 to regulate HIF1α mRNA translation, we generated five reporter vectors (Fig. 6A): EGFP reporter coding region alone; EGFP along with HIF1α 5′ UTR; EGFP along with HIF1α 3′ UTR; EGFP along with HIF1α 5′ and 3′ UTRs; and EGFP along with mutant HIF1α 3′ UTR, which lacks the RBM38-binding site in the B1 segment as showed in Fig. 5I. We showed that in p53^−/−^ HCT116 cells, knockdown of RBM38 had no effect on EGFP expression for a vector that does not carry any sequence from HIF1α transcript (Fig. 6B). Interestingly, the levels of EGFP protein were increased by 1.4-fold for the vector that carries HIF1α 5′UTR (Fig. 6C), 1.3-fold for the vector that carries HIF1α 3′UTR (Fig. 6D), and 2.2-fold for the vector that carries both HIF1α 5′ and 3′ UTRs (Fig. 6E). Most importantly, we showed that the level of EGFP protein was not significantly increased by knockdown of RBM38 for the vector that carries mutant HIF1α 3′UTR (3′UTRmut) (Fig. 6F-G, compare lanes 3-4). Again, as a control, knockdown of RBM38 by siRNA and shRNA led to increased expression of EGFP for the vector carries HIF1α 3′UTR (Fig. 6F-G, compare lanes 1-2).

### RBM38 modulates the binding of eIF4E to the cap structure on HIF1α mRNA

**Figure 7:**
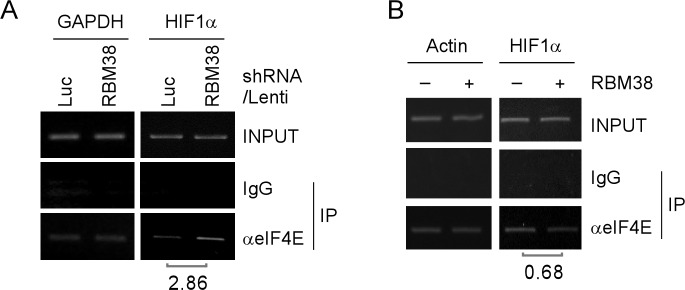
RBM38 prevents eIF4E from binding HIF1α transcript (A) p53^−/−^HCT116 cells were transduced with lentivirus particles expressing a control luciferase (Luc) shRNA or RBM38 shRNA for 3 days and then cultured at a low oxygen condition, followed by immunoprecipitation with a control IgG or anti-eIF4E. Total RNAs were purified from immunocomplexes and subjected to RT–PCR analysis to measure the level of HIF1α and GAPDH mRNAs. The relative level of HIF1α mRNA was measured by densitometry, and the relative fold change was shown below each pair. (B) p53^−/−^HCT116 cells were uninduced (−) or induced (+) to express HA-tagged RBM38 for 48 h and then treated with 500 μM CoCl_2_ for 2 hours, followed by immunoprecipitation with a control IgG or anti-eIF4E. Total RNAs were purified from immunocomplexes and subjected to RT–PCR analysis to measure the level of HIF1α and actin mRNAs. The relative level of HIF1α mRNA was measured by densitometry, and the relative fold change was shown below each pair.

To explore the mechanism by which RBM38 regulates HIF1α mRNA translation, we examined whether RBM38 modulates the binding of eIF4E, a key component of translation initiation complex eIF4F, to the cap structure of HIF1a mRNA in p53^−/−^ HCT116 cells. RNA immunoprecipitation followed by RT-PCR assay was performed and showed that the level of eIF4E associated with HIF1α mRNA was increased (2.86-fold) upon knockdown of RBM38 in p53^−/−^ HCT116 cells at a low oxygen condition (Fig. 7A). In contrast, the level of eIF4E associated with HIF1α mRNA was decreased (0.68-fold) upon ectopic expression of RBM38 in p53^−/−^ HCT 116 cells treated with 500 μM CoCl_2_ for 2 hours (Fig. 7B). These results suggest that RBM38 prevents eIF4E from binding to HIF1α transcripts, and thus inhibits HIF1α mRNA translation.

## DISCUSSION

HIF1α plays a critical role in hypoxia to improve glycolysis, oxygen delivery, and angiogenesis for tumor cells [1, 3]. Although HIF1α is mainly regulated by VHL-mediated proteasomal degradation, it can be regulated by other post-transcriptional mechanisms [16]. Indeed, we found a novel mechanism by which HIF1α expression is regulated by RBM38 via mRNA translation. We also found that RBM38 directly binds to HIF1α 5′ and 3′UTRs. Additionally, an ARE element in HIF1α 3′UTR is recognized by RBM38. Importantly, we showed that both HIF1α 5′and 3′ UTRs are necessary and sufficient for RBM38 to regulate HIF1α mRNA translation. Since RBM38 inhibits the binding of eIF4E to HIF1α cap structure, we postulate that upon binding to HIF1α 5′ and/or 3′ UTRs, RBM38 may physically hinder the binding of eIF4E to HIF1α 5′ cap structure. Alternatively, since RBM38 physically interacts with eIF4E [9]. We hypothesize that upon binding to HIF1α transcript, RBM38 and eIF4E get close together and interact with each other on the HIF1α transcript, which then prevents eIF4E from associating with HIF1α 5′-cap.

HIF1α accumulation in tumors can be induced by various stress signals, including hypoxia in tumor microenvironment, loss of a tumor suppressor, or oncogene activation [14, 19]. Increased HIF1α abundance promotes tumor growth and angiogenesis [1, 20]. In this study, we showed that RBM38 deficiency leads to increased expression of HIF1α. Thus, an obvious question would be: is there a functional connection between RBM38 and HIF1α? RBM38 is found to be overexpressed in several types of cancers [9, 21-29]. In addition, loss of RBM38 in mouse embryonic fibroblasts leads to premature senescence through activation of p53 [9]. Here, we showed that RBM38 deficiency leads to increased accumulation of HIF1α under a hypoxic condition. These results suggest that both RBM38 overexpression and deficiency lead to tumor promotion. Thus, further studies are needed to address the functional link between RBM38 and HIF1α under the hypoxic tumor microenvironment, which may explain how tumors thrive under a hypoxic condition. Additionally, since RBM38 expression and phosphorylation may be altered under a hypoxic condition, future studies are needed to address whether the binding of RBM38 to HIF1α UTRs is affected by hypoxia.

### EXPERIMENTAL PROCEDURES

### Plasmids

pGEX vector expressing GST or GST-tagged RBM38 was used for producing recombinant RBM38 protein as previously described [18]. Lentiviral vectors (pLKO.1-puro) expressing shRNA against RBM38 and luciferase were prepared as previously described [8].

To generate EGFP expression vector carrying HIF1α 5′ and/or 3′UTRs, a DNA fragment containing EGFP coding region was amplified using pEGFP-N2 vector as a template with forward primer including HA-tag, EGFP-BamHI-HA-F, and reverse primer, EGFP-R. The primers for cloning are listed in Table 1. The PCR product was digested with *Bam*HI and *Not*I and cloned into pcDNA3 vector (Invitrogen). The vector was designated as pcDNA3-HA-EGFP. A fragment containing HIF1α 5′ or 3′UTR was amplified using cDNA from H1299 cells as template with forward primer, HIF1α-5′UTR-KpnI-F or HIF1α-3′UTR-NotI-F, and reverse primer, HIF1α-5′UTR-BamHI-R or HIF1α-3′UTR-XhoI-R. The PCR products were digested with *Kpn*I and *Bam*HI for HIF1α-5UTR or *Not*I and *Xho*I for HIF1α-3′UTR and cloned into pcDNA3/HA-EGFP vector. The vectors were designated as pcDNA3/HIF1α-5′UTR/HA-EGFP and pcDNA3/HA-EGFP/HIF1α-3′UTR. To generate pcDNA3/HIF1α-5′UTR/HA-EGFP/HIF1α-3′UTR, pcDNA3/HIF1α-5′UTR/HA-EGFP vector was digested with *Kpn*I and *Bam*HI. The digested DNA fragment containing HIF1α-5′UTR was cloned into pcDNA3/HA-EGFP/HIF1α-3′UTR. The HIF1α-3′UTR deletion mutation was generated from two cDNA fragments using two-steps PCRs with HIF1α-3′UTR-NotI-F and HIF1α-3′UTR-XhoI-R, followed by subcloning into pcDNA3-HA-EGFP. The fragment 1 was amplified using primers HIF1α-3′UTR-NotI-F and HIF1α-3′UTR-mut-R whereas the fragment 2 was produced using primers HIF1α-3′UTR-mut-F and HIF1α-3′UTR-XhoI-R.

**Table 1:** Primers for RT-PCR and cloning

Primer Name	Sequence
RBM38-RT-F	5′-cgcagaaggacaccacgttcacca-3′
RBM38-RT-R	5′-tgtagtgcggggtcagcccgtct-3′
HIF1α-RT-F	5′-cacaggaaatggccttgtgaa-3′
HIF1α-RT-R	5′-ccaagcaggtcataggtggt-3′
GAPDH-RT-F	5′-agcctcaagatcatcagcaatg-3′
GAPDH-RT-R	5′-atggactgtggtcatgagtcctt-3′
HIF1α-5UT-Kpn-F	5′-gggGGTACCgcgcgcgccggcctgggcag-3′
HIF1α-5UT-Bam-R	5′-gggGGATCCGGTGAATCGGTCCCCGCGAT-3′
HIF1α-3UT-Not-F	5′-gGCGGCCGCgctttttcttaatttcattcctttttttggac actg -3′
HIF1α-3UT-Xho-R	5′-gggCTCGAGGCCTGGTCCACAGAAGATG-3′
HIF1α-3UT mut-F	CAGTAGCATCGTTTATCCCTTTTTCGAATTATTTTTAAGAAGATGCCAATATAATTTTTGTAAGAAGGC
HIF1α-3UT mut-R	GGGATAAACGATGCTACTGCAATGCAATGGTTTAAATACCAAAAAACTGAGAAAATGAG
EGFP-HA-Bam-F	5′-ggggGGATCCgccaccatgTACCCATACGATGTTCCAGATTACGCTgtgagcaagggcgaggagctg-3′
EGFP-R	5′-GTATGGCTGATTATGATCTAG-3′

### Cell culture

Human breast cancer MCF7 cell line, Human colorectal carninoma HCT116 cell line, and human non-small cell lung carcinoma H1299 cell line were obtained from the American Type Culture Collection (ATCC, Manassas, VA). p53^−/−^ HCT116 cell line was used as described [30]. Cell lines were cultured in DMEM (Invitrogen) supplemented with 10% fetal bovine serum (Hyclone) and maintained at 37°C in a humidified 5% CO_2_. p53-null HCT116 cell lines, in which RBM38 can be inducibly knocked down or in which RBM38, RBM38-S195A, or RBM38-S195D can be inducibly expressed, were generated and cultured as previously described [7, 10, 17]. Cells were subjected to hypoxia (0.1 to 1%) by exposure to 10% H_2_/5% CO_2_/balanced N_2_ at 37°C in Forma 1025/1029 Anaerobic Chamber (Thermo Scientific).

### RNA interference

For lentiviral shRNA transduction, a lentiviral vector (10 μg) expressing shRNA against luciferase or RBM38 [17], along with packaging plasmids, pRSV-REV (5 μg), pMDL g/p RRE (5 μg), and VSVG (5 μg), was cotransfected into HEK 293T cells (8 × 10^6^) with Expressfect transfection reagent (Denville Scientific). After 48 h, the supernatant containing shRNA-expressing lentiviral particles was harvested, filtered and concentrated by ultracentrifugation (25,000 rpm, 4°C, 2 h). The concentrated lentiviral particles were then used to transduce cells, followed by puromycin selection (1 μg /ml) for 3 days to remove un-transduced cells. RBM38 siRNA was used as described [9, 17].

### Western blot analysis

Cells were cultured in various conditions and whole cell lysates were prepared with 2X SDS sample buffer. Whole cell lysates were separated in 8 to 12% SDS-PAGE, transferred to a nitrocellulose membrane, and incubated with primary and secondary antibodies, followed by enhanced chemiluminescent detection. The antibodies used in this study are anti-HIF1α (BD Biosciences), anti-RBM38 (purified rabbit polyclonal), anti-p53 (monoclonal anti-serum, DO-1), and anti-Actin (Sigma).

### RNA isolation, RT-PCR

Total RNA was isolated by using Trizol reagent (Invitrogen). cDNA was synthesized using MMLV reverse transcriptase (Promega). PCR was performed with primers listed in Table 1.

### RNA-immunoprecipitation (RNA-IP)

RNA-IP was carried out as previously described [7, 31]. Briefly, cells (4 × 10^6^) were lysed with 1 ml of lysis buffer (10 mM HEPES, pH7.0, 100 mM KCl, 10 mM MgCl_2_, 0.5% NP-40, 1 mM DTT) supplemented with RiboLock Ribonuclease inhibitor (Fermentas) for 15 min on ice. Cell lysates were collected following centrifugation (13,000 rpm, 4°C, 10 min). The RNA-protein immunocomplexes were formed by incubating 0.4 ml of cell lysates with 2 μg of anti-RBM38 (purified rabbit polyclonal), anti-eIF4E (Santa Cruz, CA), or isotype control IgG at 4°C for 4 h and brought down by 20 μl of protein G bead (50% slurry). RT-PCR analysis was carried out to measure the RNA-protein interaction. The primers to amplify p21 were used as described in [9].

### Probe preparation and RNA Electrophoretic Mobility Shift Assay (REMSA)

All probes were labeled by *in vitro* transcription using a DNA fragment containing T7 promoter and various region of HIF1α 5′ or 3′UTR. Briefly, 500 ng of purified PCR product was incubated with 50 μCi of α-^32^P-UTP, 0.5 mM each of NTP (A, G, C), 20 unit of T7 RNA polymerase (Ambion) in 20 μl of reaction at 37°C for 1 h, followed by DNase I (1 unit) treatment for 15 min. The reaction mixture was purified by Sephadex G-50 column to remove unlabeled free nucleotides and the radioactivity of probes was measured by a scintillation counter. REMSA was carried out with a modified protocol as previously described [8]. Briefly, 250 nM of RBM38 recombinant protein, 100 μg/ml of yeast tRNA, and 50,000 CPM ^32^P-labeled RNA probe were mixed in 20 μl of reaction buffer (10 mM Tris-Cl, pH 8.0, 25 mM KCl, 10 mM MgCl_2_, 1 mM DTT) at 25°C for 25 min. RNA/protein complexes were digested with 100 U RNase T1 at 37°C for 15 min and then separated in 7% native PAGE gel. RNA-protein complexes were visualized by autoradiography.

### ^35^S-Metabolic labeling Assay

Cells seeded in a 6-cm plate (6 × 10^6^ cells) were washed twice with PBS and incubated in DMEM without L-methionine and L-cysteine for 1 h. Cells were then labeled with 100 μCi/ml Easy Tag EXPRESS ^35^S Protein Labeling Mix (PerkinElmer) for 30 min. Cell lysates were isolated and used for immunoprecipitation with anti-HIF1α (H1α67, Sigma) or non-immune mouse IgG. The samples from immunoprecipitation were separated in 8% SDS/PAGE. The gel was dried on 3-MM paper and the protein signals were captured by autoradiography.
